# Integrative Approaches to Interconnected Environmental Challenges: How Institutional Factors Influence Cross-Sector Integration in Dutch Rural Areas

**DOI:** 10.1007/s00267-025-02140-2

**Published:** 2025-03-07

**Authors:** Elena Bakhanova, Joanne Vinke-de Kruijf, Lara Wöhler, Beau Warbroek, Maarten Arentsen

**Affiliations:** University of Twente, Enschede, Netherlands

**Keywords:** IAD framework, Cross-sector collaboration, Integrative potential

## Abstract

Environmental challenges are increasingly often interconnected. Yet, they are commonly addressed separately, which might result in inefficiencies and missed opportunities. While it is widely acknowledged that integrative solutions can bring co-benefits and synergetic outcomes for different sectors, empirical studies that systematically explore cross-sector integration at the operational level are rare. Drawing from two case studies with a high potential for reducing the environmental impacts of energy production and agriculture while restoring nature in the Dutch rural context, this exploratory study aims to provide an improved understanding of the institutional factors that influence the fulfillment of integrative potential in rural areas. To understand how institutional factors influence integrative outcomes, we use the operational level rules that guide actions and interactions among the actors of the Institutional Analysis and Development (IAD) framework. Our analysis shows that the potential of integrative solutions is not fully recognized and realized in Dutch rural areas. Key institutional factors that hamper integration are diverging perceptions of desired outcomes (scope rule), exclusion of important actors from decision-making (position rule), and imbalances in the sharing of costs and benefits (payoff rule). We conclude that agreement on the rules of actions and interactions between the actors is necessary for fulfillment of integrative potential.

## Introduction

As the effects of climate change, biodiversity loss, and other societal challenges become more severe over time, researchers and practitioners emphasize the need to consider the interlinkages between them (Morita and Matsumoto 2018; Smith et al. 2019; Turney et al. 2020; Pörtner et al. 2021; Pettorelli et al. 2021). This is crucial since overlooking interconnectedness can result in less efficient responses and higher costs for society (Smith et al. 2019; Sharifi 2021). Such risks become more evident in highly populated regions where multiple functions have to be served by limited space (Spijkerboer et al. 2019).

These considerations have led to a growing interest in exploring, on the one hand, co-benefits and synergies and, on the other hand, conflicts and trade-offs (Klein et al. 2005; Sharifi 2020, 2021). The focus of this study is on the positive impacts that can be associated with interconnectedness, i.e. “integrative potential”. Integrative potential is defined here as “the opportunity to implement solutions that contribute to solving multiple societal challenges at the same time” [as they] “may provide co-benefits or synergies” (Warbroek et al. 2023 p. 97). Co-benefits occur when solutions proposed by actors of one sector also achieve positive effects for other sectors (Duguma et al. 2014; Kongsager 2018). Synergy, on the contrary, requires actors to focus on the bigger picture and address multiple challenges from the beginning. Compared to approaches that focus only on one challenge, an integrative approach that addresses multiple challenges is more likely to provide synergetic benefits (Sharifi 2021). Yet, in some contexts, the sectoral approach might prove to work better. For instance, when the causes and effects of a problem are bound by one sector only (Bryson et al. 2006; De Loë and Patterson 2017). Even so, consideration of integrative potential is often still highly desirable, especially in more complex contexts where multiple challenges are at stake, such as, climate adaptation and sustainable development goals (Cuevas 2016), climate adaptation and mitigation (Matocha et al. 2012), farming and water management (Whaley and Weatherhead 2015) or flood protection, hydropower and habitat loss (Metz et al. 2020).

Despite increasing interest in such cross-sector integration, there are rather limited examples of observing it in practice (Kimmich et al. 2023; Warbroek et al. 2023). An underlying problem is that current governance systems, in particular during policy implementation, are largely dominated by sectoral practices, leading to a lack of coordination and inconsistency in the priorities of different actors when it comes to integration (Spijkerboer et al. 2019; Sharifi 2021). Conversely, tackling interlinked challenges requires a situation where multiple actors from different sectors act together and agree on the overarching strategy and goals (Villamayor-Tomas et al. 2015). In this context, an institutional perspective is particularly pertinent because it draws attention to the rules that bind how the actors behave (Crawford and Ostrom 1995) as well as the conditions under which actors make their choices (Bryson et al. 2006; Duguma et al. 2014; Villamayor-Tomas et al. 2015; Runhaar et al. 2018). As such, institutions are likely to play a critical role in achieving integration in practice. Yet, few studies have explored how rules that govern actions and interactions influence achieving integrative outcomes in real life (Spijkerboer et al. 2019; Warbroek et al. 2023).

The objective of this study is to identify and better understand which and how institutional factors influence the fulfillment of integrative potential in rural areas. Empirically, we draw from two Dutch countryside cases with a high potential of tackling sustainable energy transition and other societal challenges in an integrated manner. We focus on the eastern part of the Netherlands, which has a dominating share of rural areas compared to other parts of the country and is facing significant environmental impacts from agriculture in combination with spatial demands for nature restoration, energy transition, and other societal challenges. One case study is characterized by a conflict of interest around agriculture and nature. Here, biomass production might serve as an integrative solution for sustainable energy production, nature restoration, and reduced environmental impacts and socio-economic support for the farming community. Another case study focuses on manure fermentation as an integrative solution for decreasing nitrogen pollution caused by intensive agriculture and the transition toward more sustainable energy sources. To investigate how institutional factors influence integration, we employed qualitative methods such as interviews and document analysis. In doing so, this exploratory research provides an empirical understanding of cross-sector integration at the operational level. Such understanding might be valuable for envisaging potential challenges and designing more effective processes of cross-sector collaboration among the actors.

The paper is organized as follows. First, we provide an analytical framework that guides our case study research. This is followed by an explanation of the research methods. Next, we elaborate on the results of the two case studies. The final sections discuss the findings and present our key conclusions.

## Theoretical Background

### The Role of Cross-Sector Collaboration in Fulfilling Integrative Potential

Multiple studies appeal to the need for a more integrative approach when dealing with interlinked societal challenges (Runhaar et al. 2018; Smith et al. 2019). Integration is represented by mainstreaming one sector’s objectives into the policies and practices of other sectors (Uittenbroek 2016; Runhaar et al. 2018). Such mainstreaming varies from reducing the duplication between sectoral policies to developing mutual strategies for several sectors (Willems et al. 2022). Likewise, when implementing policies, actors can plan their activities over one specific problem or over multiple societal challenges (Lyles et al. 2018). While most studies tend to focus on the system level interacting effects (e.g. Duguma et al., 2014; Berry et al. 2015), and specifically address policy integration (Di Gregorio et al. 2017; Cejudo & Trein 2023; Biesbroek, Candel 2020), this research focuses on the operational level of integration, where solutions have a physical impact. Notwithstanding these benefits, researchers also admit that integration can imply high transaction costs and may have unintended side effects due to the impossibility of predicting the risks in such a complex multi-sector setting (Bryson et al. 2006; Märker et al. 2018; Kongsager 2018; Metz et al. 2020). In addition, De Loë and Patterson (2017 p. 93) state that challenges for which causes, effects, and interests are “relatively clear, uncontentious, and bound by sector” are generally tackled most efficiently using a sectoral scope. Hence, although integration can be desirable in many cases, it is not a universal solution. This implies that the integrative potential should ideally be assessed before pursuing an integrative approach.

Integrative potential can be realized through a collaborative effort of actors representing multiple sectors. Duguma et al. (2014) claim that synergy, in particular, can hardly be achieved without cross-sector collaboration. Bryson et al. (2006 p. 44) define such collaboration as “the linking or sharing of information, resources, activities, and capabilities by organizations in two or more sectors to achieve jointly an outcome that could not be achieved by organizations in one sector separately.” While in this definition the term “sector” refers to various organizational forms (public, private, not-for-profit, and so forth), our focus is on collaboration by actors representing different problem domains (such as climate adaptation, energy, agriculture, and others).

### Institutional Analysis and Development Framework (IAD) as an Analytical Framework

Cross-sector collaboration requires looking at institutions as they determine the incentives and conditions in which actors make their choices (Bryson et al. 2006; Duguma et al. 2014; Villamayor-Tomas et al. 2015; Runhaar et al. 2018). North (1991 p. 97) defines institutions as “humanly devised constraints that structure political, economic and social interaction.” Such interaction between actors is based on formal rules (laws, property rights, policies, and so forth) and informal rules (customs, norms, and so forth) (Ostrom 2006; Grundmann and Ehlers 2016; Spijkerboer et al. 2019). In this study, we aim to understand these rules as this allows us to find out what prevents actors from different sectors from fulfilling integrative potential.

The IAD framework by Ostrom (2011) is a renowned instrument for conducting systematic institutional analysis. It was initially developed in the 1980s as an attempt to structure the elements, categories of factors, and logical interconnections that explain how institutions work (McGinnis 2011a). Since then, it has evolved significantly as well as extended in the form of other frameworks (e.g., the Social-Ecological System (SES) Framework by McGinnis and Ostrom 2014). IAD applications stretch over a diversity of problem domains, including a few examples of cross-sector integration (e.g., Villamayor-Tomas et al. 2015; Märker et al. 2018; Warbroek et al. 2023).

The core component of the IAD is an action situation, which is a space where “individuals interact, exchange goods and services, solve problems, dominate one another, and fight (among the many things that individuals do in action situations)” (Ostrom 2011 p. 11). Action situations exist within a larger context of biophysical conditions, attributes of community, and rules-in-use. Interactions among actors in an action situation lead to outcomes that are evaluated by actors through the criteria relevant to them. Outcomes, in turn, can influence the larger context and the action situation itself.

**Fig. 1:**
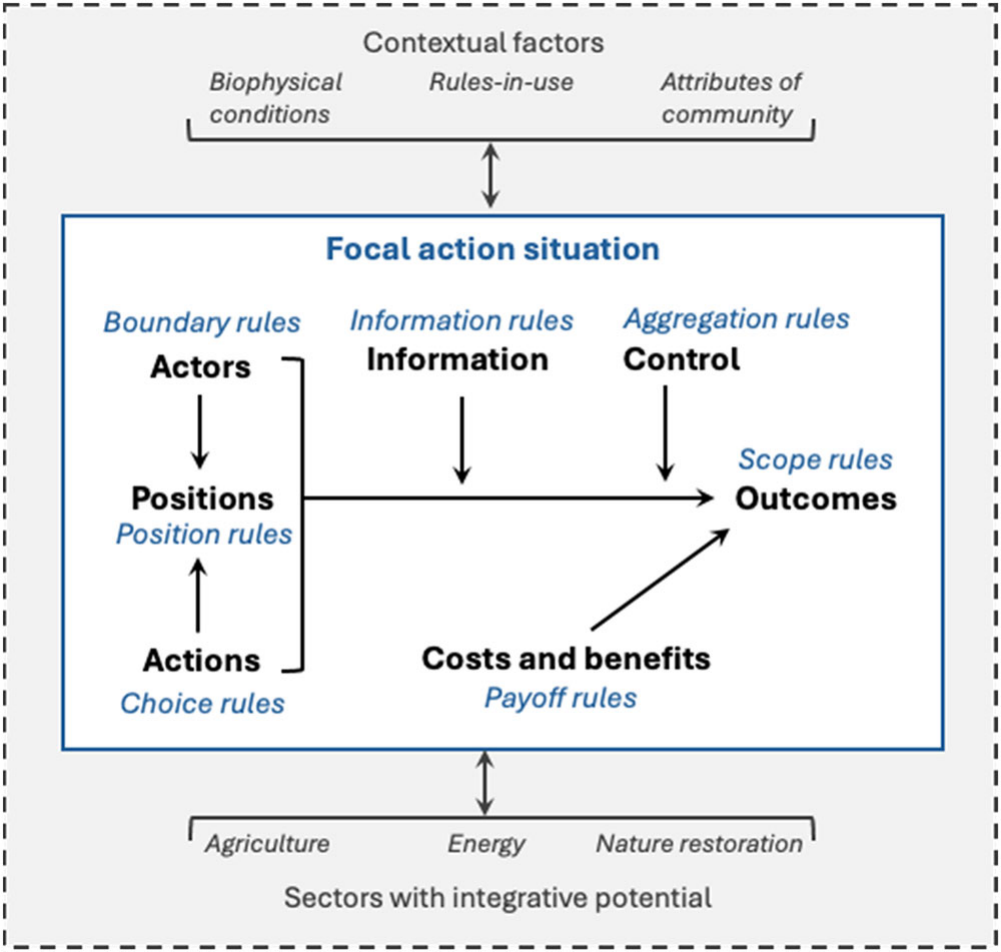
Analytical framework for the study (adapted and extended from Ostrom 2006)

According to Ostrom (2006), interactions among actors in an action situation are shaped by seven rules (Fig. 1). The scope rule represents the outcomes desired by different actors. The position and boundary rules define, respectively, who can join an action situation and under which conditions. The choice and aggregation rules represent the range of available actions and the decision-making process. Finally, information and payoff rules refer to, respectively, how information and costs are shared among actors.

Action situations and rules can be examined at different levels of choice: constitutional, collective, and operational (McGinnis 2011a). The constitutional level is defined by who is eligible to develop policies and under which rules. The collective choice level corresponds with making the rules, and the operational level matches with the implementation of on-the-ground actions (Ostrom 2011). The levels of choice provide the possibility of zooming in and out, which – depending on the analysis purpose – either helps to get to a practical level (this study) or to see a broader picture of policy context.

Applications of the IAD tend to draw attention to only one (typically sectoral) action situation. Later, the Network of Action Situations (NAS) concept was introduced to emphasize the presence of adjacent action situations that mutually influence each other. The NAS understands that “outcomes generated in one action situation help determine the rules under which interactions occur within the other action situation” (McGinnis 2011b p. 52). Warbroek et al. (2023) used the NAS concept for analyzing the Dutch energy transition at the operational level. They conclude that multiple action situations, even if interconnected, may result in non-integrative outcomes. Therefore, redesigning prevailing rules to create integrative action situations covering multiple sectors might be a way forward (Kimmich et al. 2023; Warbroek et al. 2023). Related to this, Kimmich et al. (2023) note growing research on complementarities and disconnects between action situations as part of NAS applications. Attention for institutional mechanisms to actively link action situations across sectors is hitherto missing, such as that of integrative action situations. Linkages between action situations (such as biophysical conditions, actors, flows of information and institutional processes) tend to be viewed in a dependent way, and not necessarily factors that can be consciously influenced by actors (McGinnis 2011b; Kimmich 2013; Cole et al. 2019; Ortiz-Riomalo et al. 2023). To empirically examine relevant action situations while maintaining focus, the concept of a “focal action situation” can be used. A focal action situation represents an action situation of interest that is impacted by multiple related action situations with their own groups of actors, governance, and resource units (McGinnis and Ostrom 2014). A focal action situation implies that several action situations can occur within the same context of biophysical conditions and attributes of the community (Cole et al. 2019).

To understand how prevailing rules influence the fulfillment of integrative potential in practice, this study specifically zooms into the operational choice level of the IAD framework using the concept of a focal action situation.

## Methods

This exploratory study employs qualitative case study research as the primary research strategy since we set out to understand a phenomenon that is inseparable from its context (Yin 2018). This is, we focus on the integrative potential of local settings and context-specific action situations with integrative potential that are happening in certain institutional, socio-economic, and geographical contexts. Case study research allows us to gain a sufficient level of detail on action situations and the institutional factors influencing the fulfillment of integrative potential.

We opt for a multi-case design since “analytical conclusions arising from two cases […] will be more powerful than those coming from a single case alone” (Yin 2018, p. 98). By selecting and analyzing two cases, we are able to gain a deeper understanding of the studied challenges and strengthen the validity of the study outcomes. Importantly, the case studies were not chosen for a comparative analysis.

### Case Study Selection

Our cases are selected in the context of a larger, transdisciplinary project that aimed to explore integrative approaches to the energy transition in the Overijssel province, the Netherlands. Following exploratory interviews with the key actors, we decided to select bioresources as a thematic focus. Bioresources refer here to “naturally occurring materials which are sustainably renewable and biodegradable” (Ingle et al. 2020, p. 3) and include crops, agricultural waste, forest resources, and others. Overijssel, located in the mid-east of the Netherlands, is geographically dominated by rural areas with intensive agriculture (amongst others, livestock farming) as a highly significant economic activity. Bioresources are crucial and, at the same time, contested assets due to the need to balance agriculture and nature restoration and conservation. Finding new ways of dealing with this increasing duality has been identified as an important direction where integrative approaches could lead to synergies. Energy is another sector considered in this study because of its potential for integrative bioenergy solutions. Because of their contested nature and integrative potential, bioresources are relevant to study in the Netherlands. Yet, the results are likely to be relevant to other parts of the world, for example, in areas where rainforests are exploited for agricultural and economic gain.

Having decided on the thematic focus, we made a gross list of twenty local settings with the potential of balancing bioresources between agriculture and nature. We selected our cases using the following criteria: (i) the presence of socio-environmental challenges and (ii) the presence of integrative potential provided by the availability of bioresources. The two cases that were expected to have high integrative potential were eventually selected for this study. The presence of integrative potential was verified by the research team through an additional quantitative assessment (Reijers 2021).

**Fig. 2:**
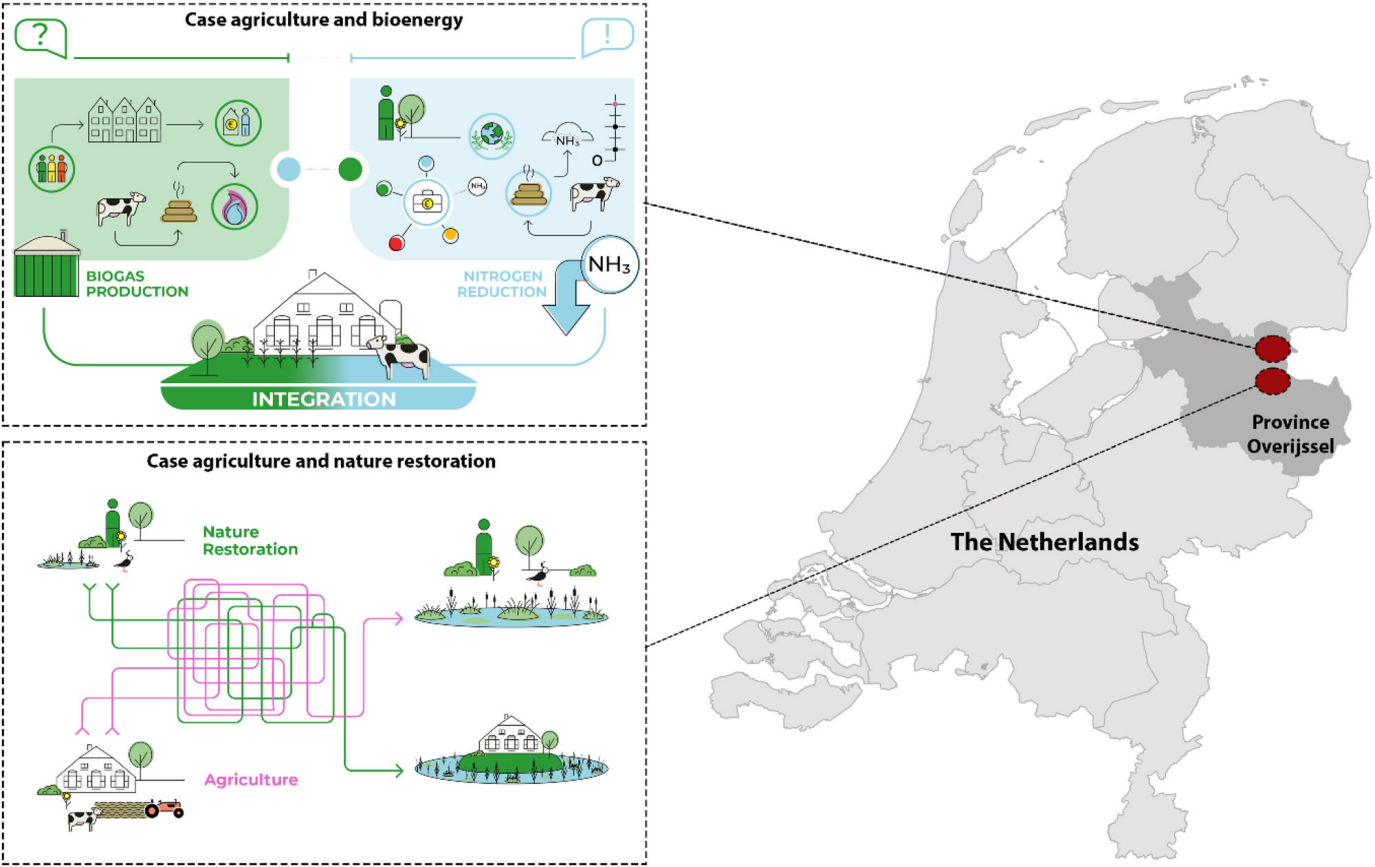
Visualization of the case study locations and key challenges

The two selected cases are visualized in Fig. 2 and referred to as: (1) Agriculture and Nature Restoration (ANR); and (2) Agriculture and Biogas (AB). Central in the ANR case are the challenges of agricultural food production and nature restoration. Integrative potential is provided by wet biomass production, which can be achieved by transforming farmland into an environmentally protected area while sustaining farmers’ income. Achieving this potential requires bringing together actors that represent nature (a societal interest) and two economic sectors (agriculture and electricity production). Central in the AB case are the challenges of agricultural food production (economic sector) and the reduction of nitrogen pollution (societal interest). Here, biogas production provides integrative potential. This potential can be captured if livestock farming is connected to gas and electricity production (economic sector). The central actors in this case are farmers and public officials.

### Data Collection and Analysis

The foundation for our case study research and data collection is provided by the basic elements of the IAD framework as defined by McGinnis (2011a). Our focus has mostly been on the rules that define interactions among actors. Data were collected through a combination of document analysis and semi-structured interviews. To guide our data collection and analysis, we prepared a case protocol which provides for each element of the IAD framework a plain language definition, a specification of what to address in our case study research and interview questions (the case protocol is available upon request). For example, a boundary rule was understood as “a specification of who is in and who is out, including why and when actors are included and/or excluded from action situations?” The interview question for this element is “When and why did the different actors start interacting around the challenge(s)?” with clarifying follow-up questions including “Were any actors excluded or did any actor leave the interactions? Why?” and “Are there mechanisms for committing actors to the ongoing process and decision-making in order to prevent them from “flying in and out”? Did these mechanisms change over time?”. The interview protocol was prepared in Dutch and also translated into English (see Supplementary Material). Overall, our questions were designed with the intention to obtain an in-depth understanding of (1) whether and to what extent actors representing relevant problem domains acknowledge the integrative potential, (2) how and under which conditions they interact with each other in a certain action situation, and (3) relevant actors’ perspectives on the outcomes and the approach to evaluating them.

To understand the legal and policy context as well as the integrative potential of the cases, we studied a variety of documents. For the ANR case, a total of ten documents were studied. This included one national and one regional policy, one formal proposal of the regional government, and six reports of studies that were conducted by consultancy companies and Universities. For the AB case, a total of fifteen documents were studied. This includes one national policy, one national study, four regional documents (two policies, one intention, one study), four local policies and five local studies. To prevent that our research impacts or interferes with ongoing, sensitive problem solving processes and to protect actors involved, we choose not to disclose detailed information about the case study locations and documents used (a detailed list of documents can be provided upon request).

We further conducted ten semi-structured interviews, which are recognized as a suitable method to obtain an in-depth understanding of actors and their interactions (Shackleton et al. 2021). We interviewed three actors for the ANR case and seven actors for the AB case. As one of the researchers, who is also a co-author of this paper, has a decade-long history of collaborating with the regional actors involved in agriculture, energy, and nature restoration activities, we had easy access to information and were able to obtain an in-depth understanding of the cases on the basis of a relatively limited number of interviews. For the ANR case, documentation (including meeting records) spanned a period of more than ten years. In addition, three representatives of relevant problem domains provided additional information during a co-design session that was part of the larger project scope.

Interviewees were selected based on (1) their role and impact on the action situation and (2) expertise. As integrative potential may concern various sectors, we tried to include perspectives from various sectors. In the ANR case, we interviewed the person leading and chairing the deliberation process in the area, an organization representing the interests of the farmers, and an organization representing the interests of the housing residents neighboring the area. In the AB case, we interviewed two public officials of the municipality (one working on energy and one on agriculture) and two of the province (one working on biogas and one on planning). We further interviewed representatives of two different energy network companies, one project developer, one farmers’ association, and one housing cooperative.

Due to Covid restrictions, the interviews took place online in the period from January to April 2022. The interviews were audio recorded and transcribed. We used an Excel-based case study template to analyze the interview results. The sections within the template corresponded with the interview questions and followed the IAD framework structure (i.e., involved actors, rules, outcomes, and evaluation criteria associated with the action situation). Our first step in data analysis was to screen the responses of different actors with the aim of finding out whether there was a significant difference in how different groups of actors interpreted the situation within each case. This helped us to define the action situation better. Then, we went through the sections of the template one by one, looking at the responses of different actors to synthesize the responses in a set of rules that bound actions and interactions between the actors. To avoid bias in the interpretation of the responses, two researchers worked with data iteratively. In addition, the researchers had different levels of familiarity with the local context and actors involved. Specifically, one of the researchers had a long history of studying energy, agriculture, and nature conservation topics in the Overijssel Province, while the other researcher brought international expertise and had no prior experience working in this specific local context. This helped to ensure a more neutral perspective while not missing out on important contextual details.

## Results

### Agriculture and Nature Restoration (ANR) Case

#### Biophysical Conditions

The case stems from the conflict of interest around the nature reserve that is surrounded by agricultural land. The landmark of the area is a raised bog; one of the few remaining in the country. Until the 1950s, the area was impacted by peat extraction. Later, it was used for buckwheat cultivation, among other agricultural activities. This negatively impacted the bog’s ecosystem, specifically through a high level of nitrogen pollution. In 2009, the nature reserve (circa 1000 hectares) was included in the list of protected Natura 2000 areas to preserve the unique ecosystem of the place. Natura 2000 is an instrument associated with the European Birds and Habitat Directives aiming for nature restoration (see www.Natura2000.nl). The Natura 2000 status of the area has potentially huge consequences for the existing farms and their agricultural food production activities since nature restoration would require a substantial increase of the water level, which would also affect the direct surroundings of the natural area. In fact, an overall restoration of the area – which would involve turning 250–400 hectares into wet or semi-wet land – makes continuation of existing agricultural food production in the proximity of the protected area impossible. At the start of our analysis, deliberations between the affected actors already lasted for more than ten years.

#### Rules-in-Use

The history of the area reconstruction included several phases. The first phase covered the period between 2005 and 2010 and represented a top-down approach. In this period, one of the bordering municipalities initiated a municipal reconstruction and implementation plan aiming at moderating bottlenecks in the area concerning agriculture, nature, water, recreation, and livability. The major result of this period was a vision document developed by public organizations and shared with the local community of farmers and citizens in a presentation and through other communication channels. During these years no significant physical outcomes were achieved.

The establishment of a Natura 2000 status initiated the next phase lasting from 2008 to 2015. Major achievements in this period were (1) preparation of a hydrological study as a base for development scenarios, (2) nitrogen analysis and mitigation measures which did not gain support in the farming community, (3) cost-benefit analysis showing that costs of nature restoration were higher than benefits, (4) investigations on the size of a buffer zone, and (5) start of ‘together works better’ approach (‘*samen werkt beter*’ in Dutch) initiated by the provincial authorities. These studies and the development of a management plan were initiated by a consortium of actors including the authorities at municipal and province levels, farming organizations, the regional water authority (Waterboard), and other organizations.

#### Attributes of Community

After the establishment of the Natura 2000 area, the farmers expressed doubts about the reliability of calculations related to nitrogen reduction targets and dissatisfaction from the consultation process, which negatively impacted trust. Additionally, lack of common understanding and reciprocity in actions of the authorities created unfavorable context for further progress. Specifically, a strong focus on environmental benefits, and a lack of consideration of the economic situation of farmers, and inappropriate options for compensating farmers’ losses were among the key concerns of the farming community. After 2015, the approach became more deliberative aiming at the inclusion of all interests in interaction processes with the ambition to reach outcomes by compromise and consensus. This change in approach helped to improve the communication process between the local authorities and the farming community. However, the difficulty in reaching an agreement on how to use the privately owned land in the semi-wet zone remained because such a solution had to simultaneously satisfy the requirements for bog preservation and provide an income for the landowners facing a decrease in productivity of their land bordering the Natura 2000 area. Hence, there was a need to explore integrative solutions and their potential further. One of the proposed solutions was to explore biomass production from such crops as reed and cattail for energy generation.

#### Rules, Evaluative Criteria, and Outcomes

The focal action situation focused on the period from 2015 and onward. The action situation evolved around the implementation of the changes in the land use from privately owned farmland to an environmentally protected area. The situation was complicated by the need to maintain farmers’ income. Specifically, in this action situation the actors interacted over the preparation of the development plan for the nature restoration area. Initially, the involved actors comprised government authorities of different levels (province, municipalities), the regional water authority, and farming associations. Later and up to the end of this study’s data acquisition, when the ‘together works better’ principle came into effect, this list was extended with local farmers, entrepreneurs, and residents (*position rule*) (Table 1). Practically, any party that expressed interest in the preparation of the development plan was allowed to join the initiative (*boundary rule*).

**Table 1:** ANR Case: Rules Defining Actions and Interactions of Actors in the Action Situation

#	Rule	Practices associated with the rule
1	Scope	Ambiguous perception of desired outcomes (improvement of nature quality, sustaining economically beneficiary food production for the farming community, house properties not affected by higher water levels in the area)
2	Position	Most interested actors, in particular residents of the neighboring areas, affected by higher water levels after nature restoration, are included in the action situation and expected to have joint responsibility for the outcome. Energy-sector actors are not yet included since wet crops are considered a poor business case for energy production.
3	Boundary	Actors are included based on an expressed interest in outcomes. Interests were diverse and extremely hard to balance and optimize. Therefore, it was important for all actors to continue being part of the process.
4	Choice	Participants gradually became acquainted with the workable and productive do’s and don’ts of the setting and the process. This was basically the work of the chair of the deliberation process.
5	Aggregation	Decision-making developed over the decade. Initially, many actions were based on resistance (among the farming community). Decision-making gradually shifted to be based on dynamic technical information and aiming for unanimity. By the end of the process, the affected farming community moved position toward complete compensation for losing land and business opportunities in favor of nature restoration
6	Information	Information was openly shared with actors inside and outside the action situation. The full openness of the process was the full merit of the chair of the deliberation process.
7	Payoff	Actors overall perceived an imbalance between private costs and public benefits. The farming community experienced a significant value conflict over public versus private benefits, since they were heavily affected by the natural restoration of the area. In the end, they gave up resistance, a position expressed in different ways during the years of deliberation.

Since the negotiations around the area have been ongoing for more than ten years, the interaction dynamics between the actors went through various phases. In the beginning, most of the actions related to the nature reserve were performed by the local authorities. The farmers had limited choices for action, although they strongly opposed the idea of creating nature reserves and semi-wet bordering zones around it. Over time, do’s and don’ts became more structured, and each actor started to have a say in the discussion over the solutions for the semi-wet zones (*choice rule*). As for information sharing, initially, the farming community expressed doubts about the reliability of technical data related to water levels, nitrogen deposition, and other associated issues (*information rule*). Later, through collaborative work, the actors managed to achieve a high level of data transparency. Still, they continued to have diverse perceptions about the project scope. For the actors, it was not fully clear whether the focus should be on increasing water level, nature recovery, or sustaining conventional farming in the existing (non-integrative) conditions (*scope rule*). In addition, actors, particularly the affected farming community, echoed strong disagreement regarding the financial benefits of the proposed nature restoration project. Since farming communities had to give up their conventional farming activities in the bordering zones, for them it has been perceived as an incompatible loss. Hence, for a long time, there was a strong imbalance between the fulfillment of public and private interests (*payoff rule*).

Once the dialog between the local authorities and farming communities started, they reached more agreement about the need to search for co-benefits across nature and socio-economic conditions for farmers. However, the evaluative criteria ranged during the decade of the project implementation from the continuation of farming without consideration of nature recovery to nature recovery with the partial continuation of farming activities or full compensation of losses for the farmers. So, for long the public interest of nature restoration clashed with the private interest of food production. The alternatives for food production, such as the production of wet biomass, were considered and studied. Yet, the business case of alternative crops – both as a partial and as an overall alternative for food production – was negative. Eventually, farmers accepted they would need to end food production in case the water would rise to restore nature and redirected their interests toward financial compensation.

Our analysis revealed that the focal action situation was non-integrative in the sense that several rules prevented it from fulfilling its integrative potential. Rules that particularly led to non-fulfillment were the scope and payoff rules. For a long time, the actors did not have an agreement on the desired outcomes (*scope rules*). This prevented them from progressing further to integrative solutions. Moreover, there was a lack of clarity on sharing the costs of such a solution (*payoff rules*), while the stakes for farmers were very high. During the interaction processes, alternative agricultural business models were explored, presented, and discussed. Wet biomass production for energy or biobased products was considered among other options. The farmers conceived the alternative business models as highly uncertain and unattractive concerning financial compensation. At the same time, our analysis further revealed that some rules became more supportive of integration over time. This was the result of a gradual and long process in which authorities and farmers tried to find common ground. For example, transparency of information, openness for other interested parties to join, and agreement on searching for unanimity in decision-making were potentially beneficial conditions for exploring (and achieving) integration.

Even though the farmers continually favored the smallest buffer zone and continuation of business as usual, in 2022 they agreed on a size of the buffer zone which would seriously affect their agricultural activities, but with the prospect of maximum overall financial compensation for loss of agricultural potential on their land. So, after many years of deliberation, farmers changed their mindset on the scope and the payoff of the process. This allowed the physical start of the reconstruction of the raised bog, among others by increasing groundwater levels in the area. By the end of the research project, the focus of the action situation was on the implementation of financial compensation for the farmers, a process which, according to several interviewees, takes another three to four years.

### Agriculture and Biogas (AB) Case

#### Biophysical conditions

The case was centered on a recently launched manure fermentation and biogas production facility. The Netherlands as a whole, and Overijssel in particular, has an intensive livestock farming sector leading to a manure surplus, i.e. manure production exceeds amounts that are allowed to be distributed as fertilizer on agricultural land according to nitrogen limits issued by EU legislation. Whilst the transportation of liquid manure is costly, the farmers have to consider other options, such as manure fermentation. Apart from the production of concentrated fertilizer for further sale, the process helps to generate energy to cover on-farm electricity and heating demand. At a larger scale, the generated biogas can be fed into the natural gas transmission system used for residential heating. In addition, timely manure collection and storage in closed containers for further fermentation helps to prevent emitting ammonium nitrogen into the air and its further deposition.

#### Rules-in-Use

According to EU and Dutch national regulation on farm-based manure management manure (RVO 2019) the farmers are in charge of dealing with manure surplus, which implies high transportation costs. Biogas production and the possibility to use it for residential heating go in line with the decree on the cessation of natural gas for heating and the use of sustainable energy sources by 2050. In addition, timely manure collection and storage in closed containers for further fermentation helps to prevent emitting ammonium nitrogen into the air and its further deposition. The reduction of ammonium emissions in farming, with dairy production taking the largest share, is an urgent topic in the Netherlands (Remkes 2022).

Despite the high potential of manure fermentation to address multiple challenges, it remains underrepresented in Dutch national energy policies. It is regarded as a solution for sustainable electricity but not for heating. This leads to fewer funding options for such projects (Warbroek et al. 2023). Manure fermentation is not part of Dutch Nitrogen policies (Rijksoverheid 2020) as a possible technology for reducing nitrogen deposition, either. This policy context is one of the factors leading to a situation where, no matter of potential benefits, biogas production from manure has not yet been developed beyond several separate projects similar to the one described in this case.

#### Rules, Evaluative Criteria, and Outcomes

The focal action situation evolved around expanding manure fermentation and biogas facilities. The actors interacted over finding feasible schemes to achieve provincial goals related to biogas production for heating and nitrogen reduction. While the interviewees mentioned heat transition and nitrogen deposition as the problem domains beneficial to integrate, the main focus remained on electricity generation from biogas facilities. Hence, an integrative action situation was in the minds of some actors, but it did not yet exist in practice (*scope rule*) (Table 2).

**Table 2:** AB Case: Rules Defining Actions and Onteractions of Actors in the Action Situation

#	Rule	Practices associated with the rule
1	Scope	Biogas production and nitrogen reduction are perceived as two separate challenges.
2	Position	Actors working on energy transition are involved in the action situation; actors working on nitrogen reduction work in a separate setting.
3	Boundary	The involvement of the actors occurs mostly due to legal obligations related to the energy transition.
4	Choice	Actions are coordinated at the level of individual proposals for biogas production plants.
5	Aggregation	Decision-making happens based on the technical and financial feasibility of the proposed individual projects for biogas production.
6	Information	Full transparency in information exchange within individual projects while only limited information is shared with external parties.
7	Payoff	The existing business model for biogas production is insufficient for the farmers to be involved on a larger scale. CO_2_ and nitrogen emissions are not financially recognized as part of it.

Energy transition with a specific focus on heating relied on the involvement of multiple actors, including province and municipality authorities, energy suppliers, grid operators, farmers, and housing cooperatives as they have specific responsibilities (*position rule*). The municipalities were perceived as the major problem owners as they were entitled to make decisions on which alternative energy sources to choose for their districts (*position and choice rules*). At the same time, they lacked instruments to support the heat transition and could not achieve unanimity between the departments responsible for energy transition, agriculture, and nitrogen pollution. Grid operators were responsible for biogas transmission through the existing gas grid; however, they had restrictions for investing in the infrastructure for the integration of biogas into the natural gas grid. Housing cooperatives, being mostly interested in ensuring affordability of the new heating options, had limited involvement in the action situation and were active mostly on cost-related matters. The participation of farmers in energy transition efforts was also rather limited. Although theoretically, this group of actors was one of the core beneficiaries of biogas production, in practice, they preferred a ‘wait-and-see’ strategy. The farmers were interested in seeing a working business case showing benefits from manure fermentation in terms of energy and fertilizer production, but associated many uncertainties with it (*payoff rule*). As a result, local authorities struggled to engage farmers in the process.

The major problem with manure and biogas projects was that there was no straightforward way to finance such projects (*payoff rule*). One interviewee noted that manure fermentation is not feasible without subsidies. National funding schemes were not always accessible as they required the financial viability of the proposed project, which was hard to ensure in the case of manure fermentation projects. Generally speaking, manure fermentation ranked low in the list of sustainable energy options. Within a farming community, small-scale farmers appeared to be in an even less favorable situation in terms of the possibility of getting funding for biogas production, showing general uncertainty for practical implementation. To sum up, the existing business model did not look convincing enough for the farmers to be involved. Additionally, despite the contribution of manure fermentation to nitrogen reduction, the avoided emissions were not depicted in any monetary form.

Since biogas production was not perceived as a highly attractive (sustainable energy) option, actor involvement in the action situation was happening largely due to legal obligations (*boundary rule*). For example, the municipality had to process applications for permits; hence, they got involved in biogas production proposals. Farmers, the grid operator, and the municipality collaboratively worked on the proposal based on its technical and financial feasibility and within the legal responsibilities that the actors had (*aggregation and choice rules*). Overall, most of the actions and decision-making happened at the level of individual projects but not at a larger scale, at which strategically connections between nitrogen reduction and energy generation could be pursued.

Similarly, information was available and transparent at the level of individual projects. Yet, information exchange did not happen between all the parties: there was an ongoing conversation between municipalities and grid operators with limited inclusion of individual farmers (*information rule*). Overall, it was not common for the farming community to disclose information widely due to privacy concerns unless data sharing was part of reporting on how public funds were used (for example, the Netherlands Enterprise Agency (*RVO - Rijksdienst voor Ondernemend Nederland* in Dutch) required the recipients of SDE funding for biogas and other renewable energy facilities to report data on yearly basis). One of the interviewees noted that it took significant time for the actors to understand the perspectives of each other and develop a shared understanding.

The expected outcome of the action situation was focused on biogas production. This excludes the reduction of nitrogen pollution as a goal. Still, some actors had a more integrated perspective and, consequently, a different set of desired outcomes in mind than others. For example, the province and large energy transmission operators desired that other actors would start recognizing the integrative potential of manure fermentation more. Evaluative criteria for the outcomes also differed among actors. Yet, the financial feasibility of manure fermentation and biogas production is seen as an important criterion by all actors.

Despite the recognition of the integrative potential by some of the actors (energy representatives of the municipality and the province and a farmer association representative), the focal action situation was non-integrative. This was visible from the scope rule: desired outcomes varied among the actors, and there was no vision of synergetic results. Such a sectoral scope rule impacted the position rule and who was involved in the action situation. Actors responsible for alleviating the nitrogen problem (especially the provincial government) did not perceive themselves being part of the action situation around the energy transition. Finally, the payoff rule and lack of financial feasibility of biogas projects led to difficulties in scaling up from individual projects to larger adoption at the level of the municipality and region.

The case reflected a strong discrepancy between the desired payoff of biogas production by manure fermentation and its actual payoff in terms of energy and financial yield and profitability. The desired payoff was predominantly expressed by public officials responsible for the local and regional energy transition, whereas the actual financial payoff was stressed by those responsible for and affected by a profitable business case, including farmers, the housing corporation and energy companies. The reducing impact of manure fermentation on ammonium and nitrogen pollution was stressed and used as extra payoff by those responsible for the energy transition. However, this integrative potential was not reflected and translated into a more profitable business case for farmers.

## Discussion

The case studies demonstrated that in a rural context, sustainable energy-related options like biomass production and manure fermentation, have the potential to contribute to reducing the environmental impacts of agriculture and to restoring nature. Despite this potential being recognized by individual actors involved in these processes, cross-sector collaboration in the form of integrative action situations is far from common practice in Dutch local and regional (energy) transition processes. When integration options were suggested during the interviews, people recognized them and confirmed their potential. At the same time, they stressed bottlenecks for their achievement. The main source of these bottlenecks is the sectoral rules that actors use when developing energy solutions that could have positive effects on connected societal challenges. Difficulties arise when actors need to find an agreement on the desired integrative outcomes, which presumes synchronizing actions with other actors as well. Thus, while integrative potential is recognized, integrative action situations are not yet the standard for addressing challenges in rural areas. Within this context, worth mentioning is that toward the end of our research project (i.e. in the Summer 2022), the Dutch government actually asked regional governments to develop area-specific and coherent plans for rural areas (*“gebiedsgerichte aanpak”* in Dutch). The aim of this approach – which has been canceled by the new government in 2024 – was to address the reduction of nitrogen emissions as well as nature, water and climate challenges in an integrated manner (Rijksoverheid 2025). Yet, such an integrative approach did not play a role in the studied cases.

Both cases addressed the challenges of more sustainable energy production, the reduction of environmental impacts in agriculture and nature restoration. Yet, actors involved had a strong preoccupation with a sectoral, task or responsibility-oriented perspective on the problem and the solution. In both cases, collaboration with the agriculture sector was key because of its position and environmental impacts and the fact that sustainability-oriented transitions in the countryside might have significant consequences for the continuation of agricultural activities. In both cases, the positions and decisions of farmers appear to be financially and economically driven. This finding is in line with international research on biogas investments and conservation behavior in agriculture (Yiridoe et al. 2009, Gebrezgabher et al. 2010; Reise et al. 2012; Ranjan et al. 2019; Veehouderij en omgeving et al. 2022). The non-economic considerations of actors in transition processes with integrative potential are strongly conditioned by institutional rules. In terms of the IAD framework, financial and economic considerations of actors are strongly connected and influenced by the scope and payoff rule, which steer toward financial viability and sectoral outcomes. In case of such an unsupportive context (stemming from sectoral rules-in-use and attributes of community), a way forward is to reconfigure action situations so that actors are facilitated to actively explore integrative potential from the outset and at the operational level, as suggested in earlier research on integrative potential (Warbroek et al. 2023). In such so-called integrative action situations, actors jointly deliberate the rules of an action situation, starting with the scope rule. Note that such an integrative action situation would by no means draw in all adjacent action situations, amounting to an all-embracing integrative focal action situation. Rather, it would involve that adjacent action situations open up to explore integrative potential thereby facilitating the emergence of a network of action situations that are mutually supportive of developing solutions for interconnected societal challenges. Here the aim is not endless integration, but to at least facilitate coordination across sectors. Overall, such an integrative rule-oriented context opens new directions for rebalancing private and social interests, with an integrative approach being one of the avenues for change.

One of the key lessons learned while working on the cases is that quantification of integrative potential (cf. Reijers 2021) can support agreement on the payoff rule among the actors. Such quantitative estimations are particularly useful when they can provide stronger arguments for initiating integrative projects by showing various benefits for each group of actors and, therefore, convincing them to collaborate. With respect to biogas production from intensive livestock farming, the quantification of the integrative potential is emerging (Aguirre-Villegas and Larson 2017; Barampouti et al. 2020; Veehouderij en omgeving et al. 2022; Veehouderij en omgeving et al. 2021a; Veehouderij en omgeving et al. 2021b; Reijers 2021). Such quantitative insights are also provided by literature on the Water-Energy-Food nexus (Medina-Santana et al. 2020; Huang et al. 2020) with recent applications becoming more practical (Huntington et al. 2021; Yuan & Lo 2022). Also relevant here is the emerging literature that focuses on the co-benefits of nature-based solutions (Raymond et al. 2017; Giordano et al. 2020). While these strands of literature commonly point to the importance of institutional design principles to facilitate integrative perspectives on interconnected societal challenges, institutional implications are seldom elaborated. Our study specifically aimed at bridging this knowledge gap.

This study faced several limitations, which provide avenues that can be explored in future research. First, in the studied cases, we focus only on those synergies that were recognized by the actors. For example, in the AB case, we investigate synergies for three sectors. This can be seen as a limitation of this study since the scope could have been extended by adding interconnections with other sectors and challenges. As very broadly scoped plans consider more challenges from the beginning, this reduces the likelihood of conflicts in the future (Lyles et al. 2018). Yet, broadening the scope and including as many challenges as possible should be done with caution. The study by Lyles et al. (2018) confirms earlier research stating that narrow-scope plans still perform better than broad-scope ones. In the ANR case, this was stressed by several interviewees; particularly by public officials responsible for sectoral challenges and actors that would be economically affected by integrative outcomes.

Another potential limitation is that, in our analysis, we specifically search for the positive effects that integrative solutions can bring. However, it could be worthwhile to critically assess the side effects, trade-offs, and potential conflicts arising from integration. Multiple researchers working on analyzing synergies and co-benefits of cross-sector integration warn to keep in mind the potential negative effects (Bryson et al. 2006; Märker et al. 2018; Kongsager 2018; Metz et al. 2020). Next, as both cases appeared to be non-integrative, they provide us with a more detailed understanding of institutional factors that hamper integration as opposed to those that support it. Still, the detailed analysis of each rule creates a foundation for diagnosing and redesigning them in a way that leads to more integrative outcomes. Looking at the cases where integrative potential has been fulfilled in practice might add value, providing a more nuanced perspective on the factors of success. This being said, we are convinced that our focus on promising integrative potential, according to the actors involved, is a very relevant starting point for researchers and practitioners who wish to understand or pursue integration.

Finally, it should be noticed that our research was limited in terms of data and scope. Our analysis and conclusion are grounded in only two cases. To understand the cases, we relied on our own contextual knowledge of the cases in combination with documents and interviews with selected key actors. To quantify integrative potential in-depth was beyond our scope. Overcoming these limitations requires research that is larger in size and disciplines involved. Based on our project, we are convinced of the need and necessity of larger, interdisciplinary projects that reveal the benefits, restrictions and the do’s and don’ts of integrative approaches to addressing multiple challenges.

## Conclusion

The importance of cross-sector integration for better responses to major environmental challenges has been acknowledged by multiple researchers and policy professionals (Runhaar et al. 2018; Smith et al. 2019; Pörtner et al. 2021; Pettorelli et al. 2021). Yet, research on how integrative potential manifests itself in practice and what factors impede its fulfillment is still limited. Drawing from two rural cases, our analysis reveals that while integrative potential is recognized, the actual realization of this potential at the operational level is hindered by prevailing rules. In fact, actors involved are rather myopic in their perspectives and actions.

The main institutional factors that hamper integration in the analyzed cases are associated with the scope rule and the payoff rule. As noted earlier by Ostrom (2006), agreement among the actors about the scope rule is crucial for further actions. In the context of cross-sector collaboration, it is even more important because sectoral or problem domain-specific perspectives on the desired outcomes lead to sectoral effects. We observed this in the AB case where a non-integrative scope rule had a direct effect on the position rule and led to the exclusion of important actors. The payoff rule represented in both cases the core of the conflict associated with financial versus economic interests. In the ANR case, we observed a clash of public (nature restoration) and private (farming) interests, while in AB case, the potential synergetic effects related to the reduction of CO2 and nitrogen emissions did not translate into financial benefits for the farmers who are expected to practically implement the integrative solution.

Especially in densely populated countries such as the Netherlands, environmental challenges are increasingly often interconnected. Our cases confirm that integrative solutions to environmental challenges can bring co-benefits and synergetic outcomes. The fulfillment of this integrative potential demands more attention for institutional factors. As the cases show, the absence of a clear set of rules that can guide actions and interactions between relevant actors of different sectors is a key barrier to achieving integrative outcomes. The overarching conclusion from both cases is that until all actors agree on the set of rules under which they operate, it is unlikely that they are able to capture existing integrative potential. A way forward could be to adjust the rules to facilitate integration in so-called integrative action situations.

From a methodological perspective, using the seven IAD rules proved to be beneficial for performing a thorough analysis of cross-sector collaboration and uncovering the source of the inability of actors to achieve synergetic outcomes. Integrative actions situations could be a means to enhance coordination amongst interconnected societal challenges and theoretically, means to purposively enhance the fulfillment of integrative potential.

## Supplementary Information

Supplementary material

## Data Availability

Data is provided within the manuscript or supplementary information files. Additional data are available upon request from the corresponding author.
