# Supplementary material for: Integrative Approaches to Interconnected Environmental Challenges: How Institutional Factors Influence Cross-Sector Integration in Dutch Rural Areas
Source: Environ Manage. 2025 Mar 7;75(5):1308–21. doi: 10.1007/s00267-025-02140-2 (PMC12033180; doi:10.1007/s00267-025-02140-2)
Supplement: Supplementary file 1 — Supplementary material [file 267_2025_2140_MOESM1_ESM.docx]

# Supplementary material

This supplementary material belongs to the manuscript “Integrative approaches to interconnected environmental challenges: How institutional factors influence cross-sector integration in Dutch rural areas” that has been prepared by Elena Bakhanova, Joanne Vinke-de Kruijf, Lara Wöhler, Beau Warbroek and Maarten Arentsen for publication in Environmental Management. This document presents an English version of the interview protocol that guided the semi-structured interviews in the two studied cases. For a Dutch version, case documentation or further information, please contact the corresponding author: Joanne Vinke-de Kruijf ([joanne.vinke@utwente.nl](mailto:joanne.vinke@utwente.nl)).

## Interview protocol

### Opening words

- Explanation of information brochure (what the research is about) and informed consent (data use, anonymity, confidentiality)
- The research is financially supported by the [*funding organisation is* *anonymized for the purposes of independent peer-review*].
- The research is conducted by different research groups of [*university is* *anonymized for the purposes of independent peer-review*] in close collaboration with the province of Overijssel and other local and regional partners
- [*Name of the project is* *anonymized for the purposes of independent peer-review*] project investigates how addressing the energy transition can be combined with other societal challenges such as sustainable neighborhoods and sustainable agriculture. For that reason we interview organizations who are involved in energy transition and/or other societal challenges.
- The aim of the interview is to learn more about the local settings in which your organization together with other organizations is working on energy transition and/or other societal challenges
- We kindly ask you to answer our questions as a representative of your organization and not as a private person
- We hope that your information will help us making suggestions for improving the ongoing energy transition in Overijssel
- Please interrupt in case my question is not clear or if I am talking too fast.
- If you permit, I would like to record the interview, which will be transcribed later on. Note that also here the confidentiality and anonymity is assured.

### Section 1: Actor’s details

In this first section I would like to ask you about your involvement in the case. In our research a case refers to an initiative, area or project where one or multiple challenges are addressed and thus different processes can be going on.

- What is your affiliation and your position in the organization?
- How and when did your organization become involved in the case?
- What was the reason that your organization became involved in the case?

### Section 2: Identification of the case

In the second section I would like to ask you about the case to understand what the case is about.

- Which challenge(s) is (are) addressed in the case?
- In case of more than one challenge, what are the reasons to combine these?
- What are the objectives with respect to the challenge(s) in the case?
- Which actions have been taken to date and what have been results?
- What are planned actions for the future?
  - Technical options and Infrastructure:
  - What technical options are considered or have been implemented to achieve the case’s objective(s)?
  - Were any technical options excluded? If yes, why?
  - What goods, resources and infrastructures are affected by the technical options you consider or have implemented?
  - Is the achievement of the case’s objective(s) affected by one or more of the following factors?
- Formal national, provincial, municipal laws and regulations
- (local) principles, routines and habits
- Traditions in local collaboration
- How participating actors perceive targets
- How actors perceive actions planned or taken
- The case’s priority for local/regional politicians

### Section 3: Identification of the current action situation

In the third section I would like to ask questions about which actors are involved and how they interact.

- - Which actors are participating in addressing the case’s challenge(s) and what is their role and responsibility? (Please mention this for every actor separately)
- In which challenge(s) does your organization participate?
- Who/What initiated the current processes around the challenge(s) your organization is involved in?
- Is there any actor dominating/leading these processes? Who? Did this change over time?
- Who is, in your perception, the problem owner of the challenge(s) in the case? Did this change over time?
  - When and why did the different actors start interacting around the challenge(s)?
- Were any actors excluded or did any actor leave the interactions? Why?
- Are there mechanisms for committing actors to the ongoing process and decision making in order to prevent them from “flying in and out”? Did these mechanisms change over time?
  - How is the information exchange between actors interacting around the challenge(s) organized?
- What (type of) information is shared and/or publicly available?
- Do all actors have equal access to all information? Why not?
- Are there any conditions by actors for the exchange of information?
- Did the information exchange change over time?
  - How is decision making organized in the interactions around the challenge(s) your organization is involved in? Specifically we want to know:
- When are decisions taken?
- Who takes decisions?
- Are any actors excluded from decision making? Why?
- Did the decision making processes change over time?
  - Who are the most relevant actors to achieve the case’s objectives?
- Why is this actor indispensable for the outcome?
- Which actor’s participation is not crucial (dispensable) for a successful outcome? Why?
- Did this relevance of actors change over time?
  - Who is having what benefits and what costs (not only monetary) if the case’s objectives are achieved?
- Is it clear to all how the costs are covered and distributed? Do all participants agree about this distribution of costs? If not, why?
- When was the first time the costs have been discussed in the process?
- Is there a direct connection between who is considered problem owner and the distribution of costs?
- Did the distribution of costs and benefits change over time?
  - Initially you outlined the case’s objective. Is there full agreement about the objective among all actors?
- What different perceptions did/do exist about the objective?
- When should the outcome be achieved?
- Did the objective or perceptions thereof change over time?

### Section 4: Assessment of the case’s integrative potential

As a next step, we would like to ask a few questions specifically about combining different challenges. With combining we imply working on different challenges at the same time.

- - Is the case considering combining the energy transition with one or more of the following societal challenges?
- Climate adaptation like, more space for water and water retention
- Social inclusion of inhabitants
- Biodiversity
- Sustainable agriculture
- Infrastructure revision
- Other…

*[An answer to this question might have already been (partially) given in section 2. If this is the case, indicate here and ask for any potential addition]*

- - What are you expecting from the effort to combine activities to address different challenges?
- What could be added values?
- What could be tradeoffs?
  - Did you or any other actor do any exploratory study or analysis to investigate whether combining challenges would be of added value? If yes, what was the outcome of this?

### Section 5: Requirements for an integrative action situation

Finally, we would like to ask a few questions about the changes needed to (further) combine the different challenges. Here we are interested in your organization’s opinion/perspective on required changes.

- Given that the energy transition would be combined with one or more other societal challenge, how would you define the objective of such a combined effort?
- Would the objective differ from the current one?
- When in your perception has the combined effort been successfully achieved?
- What are concrete indicators for successful combined outcomes?
- Do you foresee any hurdles? If yes, which ones?
  - Does a combined addressing of different challenges require changes in the role and position of the actors currently involved?
- How would their role need to change?
- Should one actor lead/ dominate the combined effort of addressing the challenges? Why?
- Should additional actors, who are currently not involved, participate?
- Should actors who are currently involved be excluded?
  - Are changes for one or more of the following aspects required in order to change from the current setting to addressing the challenges combinedly:
- The way information is exchanged
- The type of information
- The public availability of information
- The current decision making mechanisms
- The current sharing of the costs and benefits among the participating organizations

### Closing words

- - These were my questions. Do you have any remaining comments or questions?
  - Finally, I kindly thank you for your cooperation and would like to stress once more that your information will stay strictly confidential.
  - If you wish, we are happy to share both, the scientific as well as practice-oriented outputs with you.
